# Rab35 promotes the recruitment of Rab8, Rab13 and Rab36 to recycling endosomes through MICAL-L1 during neurite outgrowth

**DOI:** 10.1242/bio.20148771

**Published:** 2014-08-01

**Authors:** Hotaka Kobayashi, Kan Etoh, Norihiko Ohbayashi, Mitsunori Fukuda

**Affiliations:** Laboratory of Membrane Trafficking Mechanisms, Department of Developmental Biology and Neurosciences, Graduate School of Life Sciences, Tohoku University, Aobayama, Aoba-ku, Sendai, Miyagi 980-8578, Japan

**Keywords:** Rab35, MICAL-L1, Recycling endosome, Neurite outgrowth

## Abstract

Small GTPase Rab35 is an important molecular switch for endocytic recycling that regulates various cellular processes, including cytokinesis, cell migration, and neurite outgrowth. We previously showed that active Rab35 promotes nerve growth factor (NGF)-induced neurite outgrowth of PC12 cells by recruiting MICAL-L1, a multiple Rab-binding protein, to Arf6-positive recycling endosomes. However, the physiological significance of the multiple Rab-binding ability of MICAL-L1 during neurite outgrowth remained completely unknown. Here we report that Rab35 and MICAL-L1 promote the recruitment of Rab8, Rab13, and Rab36 to Arf6-positive recycling endosomes during neurite outgrowth. We found that Rab35 functions as a master Rab that determines the intracellular localization of MICAL-L1, which in turn functions as a scaffold for Rab8, Rab13, and Rab36. We further showed by functional ablation experiments that each of these downstream Rabs regulates neurite outgrowth in a non-redundant manner downstream of Rab35 and MICAL-L1, e.g. by showing that knockdown of Rab36 inhibited recruitment of Rab36-specific effector JIP4 to Arf6-positive recycling endosomes, and caused inhibition of neurite outgrowth without affecting accumulation of Rab8 and Rab13 in the same Arf6-positive area. Our findings suggest the existence of a novel mechanism that recruits multiple Rab proteins at the Arf6-positive compartment by MICAL-L1.

## INTRODUCTION

Membrane trafficking, which is a process that transports proteins and lipids from one organelle to another organelle by means of vesicular carriers, is fundamental to various cellular processes, including cell division, signal transduction, and neuronal functions (Maxfield and McGraw, 2004; Grant and Donaldson, 2009; Sorkin and von Zastrow, 2009; Scita and Di Fiore, 2010). Rab family small GTPases are conserved intracellular molecular switches that regulate membrane trafficking (Zerial and McBride, 2001). In mammals, the Rab family consists of approximately 60 isoforms that exhibit isoform-specific intracellular localization (Fukuda, 2008; Stenmark, 2009). Rabs cycle between a GTP-bound active form and a GDP-bound inactive form, and active Rabs promote membrane trafficking by recruiting specific effector molecules, including lipid kinases, phosphatases, coat adaptors, BAR proteins, kinesin motor adaptors, dynamin adaptors, myosin motors, actin regulatory proteins, tethering factors, and phospholipid binding proteins. Because each Rab isoform has a distinct effector-binding property (Fukuda et al., 2008; Kanno et al., 2010), the output of each Rab-signaling is unique and isoform-specific.

Rab35 is a molecular switch for endocytic recycling and regulates various cellular processes, including cytokinesis, cell migration, and neurite outgrowth (Kouranti et al., 2006; Chevallier et al., 2009; Shim et al., 2010). We previously reported finding that active Rab35 dramatically promotes nerve growth factor (NGF)-induced neurite outgrowth of PC12 cells (Kanno et al., 2010; Fukuda et al., 2011). In response to NGF stimulation, Rab35 localizes to Arf6-positive recycling endosomes and recruits Rab35 effector MICAL-L1, whose presence is required for neurite outgrowth of PC12 cells (Kobayashi and Fukuda, 2012; Kobayashi and Fukuda, 2013a; Kobayashi and Fukuda, 2013b). Interestingly, MICAL-L1 also serves as a scaffold protein for EHD1 on recycling endosomes (Sharma et al., 2009; Kobayashi and Fukuda, 2013a; Giridharan et al., 2013) and interacts with other Rabs besides Rab35, i.e. with Rab8A, Rab8B, Rab10, Rab13, Rab15, and Rab36 (Fukuda et al., 2008; Sharma et al., 2009; Rahajeng et al., 2012). However, the physiological significance of the multiple Rab binding ability of MICAL-L1 during neurite outgrowth remained completely unknown.

Here we report finding that Rab35 concentrates multiple Rabs, i.e. Rab8, Rab13, and Rab36, at Arf6-positive recycling endosomes through MICAL-L1 during NGF-induced neurite outgrowth of PC12 cells. Rab35 functions as a master Rab that recruits MICAL-L1 to Arf6-positive recycling endosomes in response to NGF stimulation, consistent with our previous finding (Kobayashi and Fukuda, 2013a). The MICAL-L1 in turn recruits Rab8, Rab13, and Rab36 to the same compartment during neurite outgrowth of PC12 cells, i.e. MICAL-L1 functions as an effector for Rab35 and as a scaffold for Rab8, Rab13, and Rab36 in PC12 cells. On the basis of our findings, we propose a novel Rab mechanism that is triggered by a master Rab and a multiple-Rab-interacting molecule and concentrates multiple Rabs at the same membrane compartment.

## RESULTS

### Rab35, not Rab8, Rab13, or Rab36, recruits MICAL-L1 to Arf6-positive recycling endosomes

**Fig. 1.**
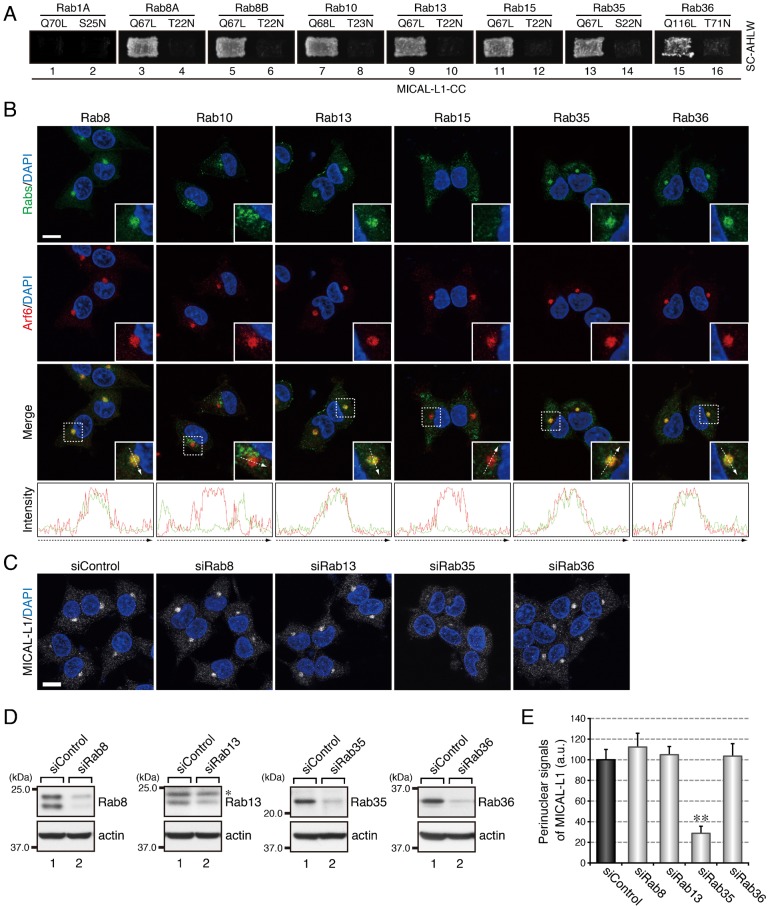
Rab35 functions as a master Rab that determines the intracellular localization of MICAL-L1. (A) Active form-dependent interaction of Rab8A, Rab8B, Rab10, Rab13, Rab15, Rab35, and Rab36 with MICAL-L1-CC. Yeast two-hybrid assays were performed to investigate whether the Rabs indicated interacted with MICAL-L1-CC. Rab1A is a negative control that does not bind MICAL-L1 at all (Fukuda et al., 2008). (B) Colocalization of Rab8, Rab13, Rab35, and Rab36 with Arf6 in PC12 cells. After stimulating PC12 cells with NGF for 6 hr, the cells were fixed and stained with the anti-Rab antibodies indicated, anti-Arf6 antibody, and DAPI. The insets are magnified views of the boxed areas. Fluorescence intensity along the broken arrows is shown at the bottom. (C) Disappearance of MICAL-L1 signals from the perinuclear area of PC12 cells after depleting them of Rab35. PC12 cells were treated with siControl, siRab8 (siRab8A + siRab8B), siRab13, siRab35, or siRab36, and after stimulating the cells with NGF for 6 hr, the cells were fixed and stained with anti-MICAL-L1 antibody and DAPI. Scale bars: 10 µm. (D) Reduced expression of Rab8, Rab13, Rab35, and Rab36 in the cell treated with siRab8, siRab13, siRab35, and siRab36, respectively. Cell lysates of PC12 cells treated with siRNAs were immunoblotted with the anti-Rab antibodies indicated and anti-actin antibody. The asterisk indicates a non-specific band of the anti-Rab13 antibody. (E) Perinuclear MICAL-L1 signals (mean and SE; arbitrary units, a.u.) of siControl-treated, siRab8-treated, siRab13-treated, siRab35-treated, and siRab36-treated PC12 cells after stimulating the cells with NGF for 6 hr (n = 60 from 3 independent experiments).

We previously reported finding that when PC12 cells are stimulated with NGF, Rab35 recruits its effector molecule MICAL-L1 to Arf6-positive recycling endosomes (Kobayashi and Fukuda, 2013a). Because MICAL-L1 has been shown to interact with multiple other Rabs besides Rab35, i.e. with Rab8A, Rab8B, Rab10, Rab13, Rab15, and Rab36 (Fukuda et al., 2008), we hypothesized that MICAL-L1 also functions as an effector molecule for multiple Rabs during neurite outgrowth. To test our hypothesis, first we performed by yeast two-hybrid assays to determine whether MICAL-L1 specifically interacts with active forms of any other Rabs. The results showed that MICAL-L1 interacts with constitutively active mutants (Rab8A-Q67L, Rab8B-Q67L, Rab10-Q68L, Rab13-Q67L, Rab15-Q67L, and Rab36-Q116L), but not with their constitutively inactive mutants (Rab8A-T22N, Rab8B-T22N, Rab10-T23N, Rab13-T22N, Rab15-T22N, and Rab36-T71N), the same as it specifically recognized active Rab35 (Fig. 1A), suggesting that MICAL-L1 may function as an effector molecule for these other Rabs.

Because Rab effectors generally localize to specific intracellular compartments through interaction with their partner Rabs (Stenmark, 2009), we next investigated whether these MICAL-L1-binding Rabs localize at Arf6-positive recycling endosomes in NGF-stimulated PC12 cells, the same as MICAL-L1 does. To do so, we performed immunofluorescence analyses, and the results showed that Rab8, Rab13, Rab35, and Rab36 localized at Arf6-positive recycling endosomes, the same as MICAL-L1 does (Fig. 1B). Rab10, on the other hand, localized in the perinuclear area away from Arf6-positive recycling endosomes and Rab15 localized in the peripheral region of the cells (Fig. 1B). Thus, it appeared possible that Rab8, Rab13, and Rab36 recruit MICAL-L1 to the Arf6-positive compartment, the same as Rab35 does. To investigate this possibility, we depleted each of these candidate Rabs alone by RNA interference and analyzed the intracellular localization of MICAL-L1 in NGF-stimulated PC12 cells. To our surprise, however, depletion of Rab8 (Rab8A + Rab8B), Rab13, or Rab36 did not alter the localization of MICAL-L1 (Fig. 1C–E), even though depletion of Rab35 dramatically reduced the perinuclear MICAL-L1 signals, the same as reported previously (Kobayashi and Fukuda, 2013a). We therefore concluded that Rab35 is the sole Rab isoform that recruits MICAL-L1 to Arf6-positive recycling endosomes and that the interactions of MICAL-L1 with Rab8, Rab13, and Rab36 may have other functions during neurite outgrowth.

### MICAL-L1 that has interacted with Rab35 is able to interact with Rab8A, Rab8B, Rab13, and Rab36

**Fig. 2.**
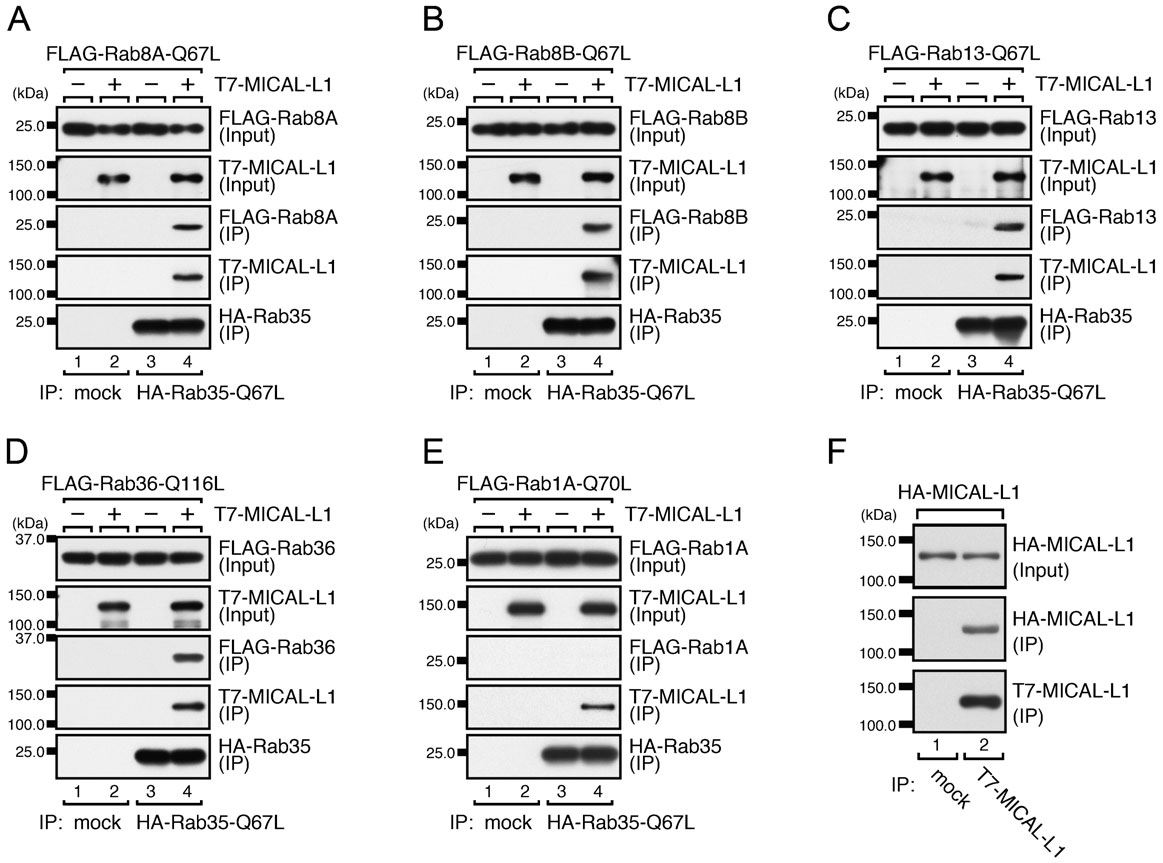
MICAL-L1 that has interacted with Rab35 then interacts with Rab8A, Rab8B, Rab13, and Rab36. (A–E) Interaction of Rab35 with Rab8A (A), Rab8B (B), Rab13 (C), and Rab36 (D), but not with Rab1A (E), mediated by MICAL-L1. Coimmunoprecipitation assays were performed to investigate whether HA–Rab35-Q67L would interact with FLAG–Rab8A-Q67L (A), FLAG–Rab8B-Q67L (B), FLAG–Rab13-Q67L (C), FLAG–Rab36-Q116L (D), or FLAG–Rab1A-Q70L (E) in the absence or presence of T7–MICAL-L1. (F) Dimerization of MICAL-L1. Coimmunoprecipitation assays were performed to investigate whether HA–MICAL-L1 would interact with T7–MICAL-L1.

Since neither Rab8, Rab13, nor Rab36 recruited MICAL-L1 to Arf6-positive recycling endosomes and MICAL-L1 has been shown to be required for tubular endosomal localization of Rab8 in HeLa cells (Sharma et al., 2009), we rejected our initial hypothesis and instead hypothesized the existence of another mechanism in which a Rab effector (MICAL-L1) recruits its multiple Rabs (Rab8, Rab13, and Rab36) to a specific membrane compartment (recycling endosomes). If this mechanism existed, MICAL-L1 would need to simultaneously interact with at least two Rabs (Rab35 plus Rab8, Rab13, or Rab36), because MICAL-L1 is recruited to Rab35 at Arf6-positive recycling endosomes (Kobayashi and Fukuda, 2013a) and Rab8, Rab13, and Rab36 are recruited to MICAL-L1 in the same compartment. To test our new hypothesis biochemically, we performed coimmunoprecipitation assays to investigate whether MICAL-L1 that has interacted with Rab35 is able to interact with Rab8A, Rab8B, Rab13, and Rab36. The results showed that Rab35 interacted with MICAL-L1 and that MICAL-L1 then interacted with Rab8A, Rab8B, Rab13, or Rab36 (Fig. 2A–D), indicating that MICAL-L1 is able to simultaneously interact with Rab35 and with Rab8A, Rab8B, Rab13, and/or Rab36. By contrast, Rab1A, which does not bind MICAL-L1 (Fig. 1A), did not coimmunoprecipitate with Rab35 even in the presence of MICAL-L1 (Fig. 2E). We also planned to detect a complex between MICAL-L1 and multiple Rabs at the endogenous protein level, but we were unable to do so because of the unavailability of antibodies that can be used for coimmunoprecipitation experiments. Since we discovered that MICAL-L1 forms a dimer (Fig. 2F), MICAL-L1 is likely to interact with multiple Rabs simultaneously through dimerization or oligomerization.

### Hierarchy of Rabs recruited to Arf6-positive recycling endosomes in response to NGF stimulation: Rab35 and MICAL-L1 recruit Rab8, Rab13, and Rab36 to the Arf6-positive recycling endosomes

**Fig. 3.**
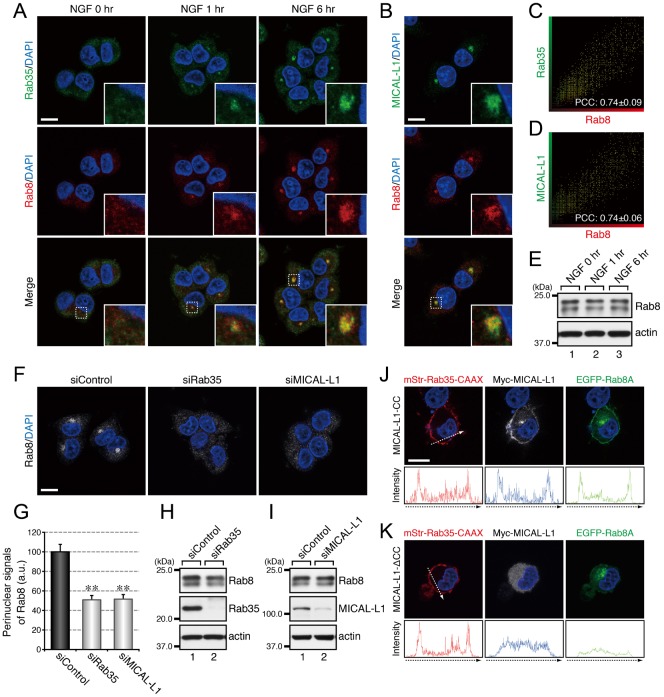
Rab35 and MICAL-L1 recruit Rab8 to recycling endosomes. (A) Accumulation of Rab35 and Rab8 in the perinuclear area of PC12 cells in response to NGF stimulation. After NGF stimulation for 0 hr, 1 hr, and 6 hr, PC12 cells were fixed and stained with anti-Rab35 antibody, anti-Rab8 antibody, and DAPI. (B) Colocalization between MICAL-L1 and Rab8 in PC12 cells. After NGF stimulation for 6 hr PC12 cells were fixed and stained with anti-MICAL-L1 antibody, anti-Rab8 antibody, and DAPI. The insets in panels A and B are magnified views of the boxed areas. (C,D) Intensity scatter plot of Rab35 signals versus Rab8 signals (C) and of MICAL-L1 signals versus Rab8 signals (D) in PC12 cells after NGF stimulation for 6 hr. The Pearson's correlation coefficient (PCC) value (mean ± SD) for the relation between them is shown at the bottom (n = 30 from 3 independent experiments). (E) Unaltered expression of Rab8 in PC12 cells during NGF stimulation. After NGF stimulation of PC12 cells for 0 hr, 1 hr, and 6 hr, cell lysates were immunoblotted with anti-Rab8 antibody and anti-actin antibody. (F) Disappearance of Rab8 signals from the perinuclear area of Rab35-depleted and MICAL-L1-depleted PC12 cells. After NGF stimulation for 6 hr PC12 cells treated with siControl, siRab35, or siMICAL-L1 were fixed and stained with anti-Rab8 antibody and DAPI. (G) Perinuclear Rab8 signals (mean and SE) of siControl-treated, siRab35-treated, and siMICAL-L1-treated PC12 cells after NGF stimulation for 6 hr (n = 60 from 3 independent experiments). (H,I) Unaltered expression of Rab8 in Rab35-depleted PC12 cells (H) and MICAL-L1-depleted PC12 cells (I). Cell lysates of PC12 cells treated with siControl, siRab35, or siMICAL-L1 were immunoblotted with anti-Rab8 antibody and anti-actin antibody. (J,K) Translocation of Rab8 to the plasma membrane after expression of Rab35 and MICAL-L1 at the plasma membrane. After NGF stimulation for 6 hr PC12 cells transiently expressing mStr–Rab35-Q67L–CAAX and EGFP–Rab8A-Q67L together with Myc–MICAL-L1-CC (J) or Myc–MICAL-L1-ΔCC (K) were fixed and stained with anti-Myc tag antibody and DAPI. Fluorescence intensity along the broken arrows is shown at the bottom. Scale bars: 10 µm.

**Fig. 4.**
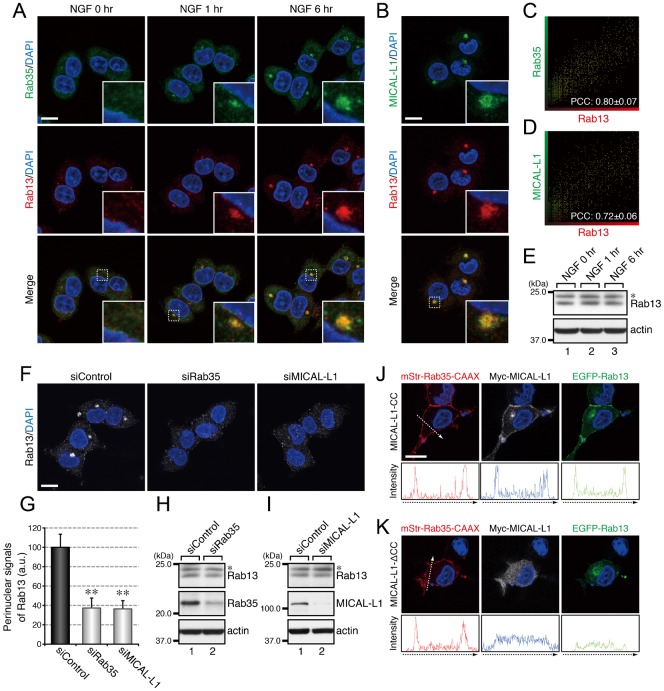
Rab35 and MICAL-L1 recruit Rab13 to recycling endosomes. (A) Accumulation of Rab35 and Rab13 in the perinuclear area of PC12 cells in response to NGF stimulation. After NGF stimulation for 0 hr, 1 hr, and 6 hr, PC12 cells were fixed and stained with anti-Rab35 antibody, anti-Rab13 antibody, and DAPI. (B) Colocalization between MICAL-L1 and Rab13 in PC12 cells. After NGF stimulation for 6 hr PC12 cells were fixed and stained with anti-MICAL-L1 antibody, anti-Rab13 antibody, and DAPI. The insets in panels A and B are magnified views of the boxed areas. (C,D) Intensity scatter plot of Rab35 signals versus Rab13 signals (C) and of MICAL-L1 signals versus Rab13 signals (D) in PC12 cells after NGF stimulation for 6 hr. The Pearson's correlation coefficient (PCC) value (mean ± SD) for the relation between them is shown at the bottom (n = 30 from 3 independent experiments). (E) Unaltered expression of Rab13 in PC12 cells during NGF stimulation. After NGF stimulation of PC12 cells for 0 hr, 1 hr, and 6 hr, cell lysates were immunoblotted with anti-Rab13 antibody and anti-actin antibody. (F) Disappearance of Rab13 signals from the perinuclear area of Rab35-depleted and MICAL-L1-depleted PC12 cells. After NGF stimulation for 6 hr PC12 cells treated with siControl, siRab35, or siMICAL-L1 were fixed and stained with anti-Rab13 antibody and DAPI. (G) Perinuclear Rab13 signals (mean and SE) of siControl-treated, siRab35-treated, and siMICAL-L1-treated PC12 cells after NGF stimulation for 6 hr (n = 60 from 3 independent experiments). (H,I) Unaltered expression of Rab13 in Rab35-depleted PC12 cells (H) and MICAL-L1-depleted PC12 cells (I). Cell lysates of PC12 cells treated with siControl, siRab35, or siMICAL-L1 were immunoblotted with anti-Rab13 antibody and anti-actin antibody. The asterisks indicate a non-specific band of the anti-Rab13 antibody. (J,K) Translocation of Rab13 to the plasma membrane after expression of Rab35 and MICAL-L1 at the plasma membrane. After NGF stimulation for 6 hr PC12 cells transiently expressing mStr–Rab35-Q67L–CAAX and EGFP–Rab13-Q67L together with Myc–MICAL-L1-CC (J) or Myc–MICAL-L1-ΔCC (K) were fixed and stained with anti-Myc tag antibody and DAPI. Fluorescence intensity along the broken arrows is shown at the bottom. Scale bars: 10 µm.

**Fig. 5.**
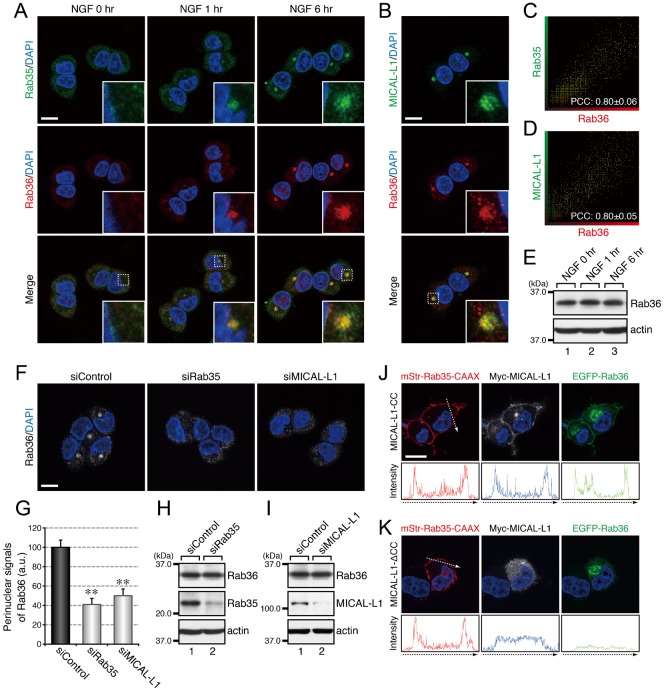
Rab35 and MICAL-L1 recruit Rab36 to recycling endosomes. (A) Accumulation of Rab35 and Rab36 in the perinuclear area of PC12 cells in response to NGF stimulation. After NGF stimulation for 0 hr, 1 hr, and 6 hr, PC12 cells were fixed and stained with anti-Rab35 antibody, anti-Rab36 antibody, and DAPI. (B) Colocalization between MICAL-L1 and Rab36 in PC12 cells. After NGF stimulation for 6 hr PC12 cells were fixed and stained with anti-MICAL-L1 antibody, anti-Rab36 antibody, and DAPI. The insets in panels A and B are magnified views of the boxed areas. (C,D) Intensity scatter plot of Rab35 signals versus Rab36 signals (C) and of MICAL-L1 signals versus Rab36 signals (D) in PC12 cells after NGF stimulation for 6 hr. The Pearson's correlation coefficient (PCC) value (mean ± SD) for the relation between them is shown at the bottom (n = 30 from 3 independent experiments). (E) Unaltered expression of Rab36 in PC12 cells during NGF stimulation. After NGF stimulation for 0 hr, 1 hr, and 6 hr, cell lysates of PC12 cells were immunoblotted with anti-Rab36 antibody and anti-actin antibody. (F) Disappearance of Rab36 signals from the perinuclear area of Rab35-depleted and MICAL-L1-depleted PC12 cells. After NGF stimulation for 6 hr PC12 cells treated with siControl, siRab35, or siMICAL-L1 were fixed and stained with anti-Rab36 antibody and DAPI. (G) Perinuclear Rab36 signals (mean and SE) of siControl-treated, siRab35-treated, and siMICAL-L1-treated PC12 cells after NGF stimulation for 6 hr (n = 60 from 3 independent experiments). (H,I) Unaltered expression of Rab36 in Rab35-depleted PC12 cells (H) and MICAL-L1-depleted PC12 cells (I). Cell lysates of PC12 cells treated with siControl, siRab35, or siMICAL-L1 were immunoblotted with anti-Rab36 antibody and anti-actin antibody. (J,K) Translocation of Rab36 to the plasma membrane after expression of Rab35 and MICAL-L1 at the plasma membrane. After NGF stimulation for 6 hr PC12 cells transiently expressing mStr–Rab35-Q67L–CAAX and EGFP–Rab36-Q116L together with Myc–MICAL-L1-CC (J) or Myc–MICAL-L1-ΔCC (K) were fixed and stained with anti-Myc tag antibody and DAPI. Fluorescence intensity along the broken arrows is shown at the bottom. Scale bars: 10 µm.

Since we previously reported finding that Rab35 and MICAL-L1 concomitantly accumulate at Arf6-positive recycling endosomes when PC12 cells are stimulated with NGF (Kobayashi and Fukuda, 2013a), we investigated the subcellular localization of Rab8, Rab13, and Rab36 in NGF-stimulated PC12 cells and in control PC12 cells. The results of immunofluorescence analyses showed that Rab8, Rab13, and Rab36 also accumulated in the perinuclear area in response to NGF stimulation and that they colocalized with both Rab35 and MICAL-L1 (Fig. 3A–D, Fig. 4A–D, Fig. 5A–D). The increases in perinuclear Rab8, Rab13, and Rab36 signals were unlikely to be attributable to their increased expression in response to NGF stimulation, because their levels of expression before and after NGF stimulation were similar (Fig. 3E, Fig. 4E, Fig. 5E).

Because four different Rabs, i.e. Rab8, Rab13, Rab35, and Rab36, accumulated at the same Arf6-positive recycling endosomes, we performed RNA interference (RNAi)-mediated knockdown experiments combined with immunofluorescence analyses in an attempt to determine the hierarchy of the Rabs recruited to this compartment. When Rab35 or MICAL-L1 was depleted by RNAi, there was a dramatic reduction in the perinuclear signals of Rab8, Rab13, and Rab36 (Fig. 3F,G, Fig. 4F,G, Fig. 5F,G), indicating that both Rab35 and MICAL-L1 are required for the perinuclear recruitment of Rab8, Rab13, and Rab36. Since the level of expression of these Rabs were unaltered by the depletion of Rab35 and MICAL-L1 (Fig. 3H,I, Fig. 4H,I, Fig. 5H,I), the Rab8, Rab13, and Rab36 in the Rab35-depleted or MICAL-L1-depleted PC12 cells were likely to have been dispersed from the perinuclear compartments into the cytosol or other membrane compartments. Depletion of Rab8, Rab13, or Rab36 alone, on the other hand, did not alter the perinuclear signals of either of the other two Rabs (supplementary material Fig. S1A–C), e.g. depletion of Rab8 did not alter the perinuclear signals of Rab13 or Rab36 (supplementary material Fig. S1A).

If Rab35 and its effector MICAL-L1 primarily determine the perinuclear localization of Rab8, Rab13, and Rab36, then ectopically expressing Rab35 and MICAL-L1 at different membrane compartments should alter the intracellular localization of these Rabs, and we used a plasma membrane-targeting sequence (CAAX) of K-Ras (Hancock et al., 1991) to determine whether ectopic expression of Rab35 and MICAL-L1 actually does alter the intracellular localization of Rab8, Rab13, and Rab36. The results showed that Rab8A, Rab8B, Rab13, and Rab36 were translocated to the plasma membrane in Rab35-Q67L–CAAX and MICAL-L1-CC-expressing PC12 cells (Fig. 3J, Fig. 4J, Fig. 5J). By contrast, expression of Rab35-Q67L–CAAX together with MICAL-L1-ΔCC (a Rab-binding-deficient mutant of MICAL-L1) (Kobayashi and Fukuda, 2013a) did not result in translocation of these Rabs to the plasma membrane (Fig. 3K, Fig. 4K, Fig. 5K), thereby demonstrating that Rab35 determines the intracellular localization of Rab8, Rab13, and Rab36 through MICAL-L1.

### Rab8, Rab13, and Rab36 function downstream of Rab35 and MICAL-L1 as essential mediators for neurite outgrowth

In the next set of experiments, we investigated whether Rab8, Rab13, and Rab36, all of which are recruited to Arf6-positive recycling endosomes by Rab35 and MICAL-L1, are also involved in NGF-induced neurite outgrowth of PC12 cells, as are Rab35 and MICAL-L1 (Kobayashi and Fukuda, 2012; Kobayashi and Fukuda, 2013a). Intriguingly, depleting PC12 cells of Rab8, Rab13, or Rab36 alone was sufficient to inhibit NGF-induced neurite outgrowth (supplementary material Fig. S2A,C), and functional involvement of Rab8, Rab13, and Rab36 in neurite outgrowth was confirmed by a dominant negative approach in which their constitutively inactive mutants were used. Consistent with the results of knockdown experiments, expression of Rab8-T22N (Rab8A-T22N + Rab8B-T22N), Rab13-T22N, or Rab36-T71N alone inhibited NGF-induced neurite outgrowth of PC12 cells (supplementary material Fig. S2B,D), the same as expression of Rab35-S22N did.

**Fig. 6.**
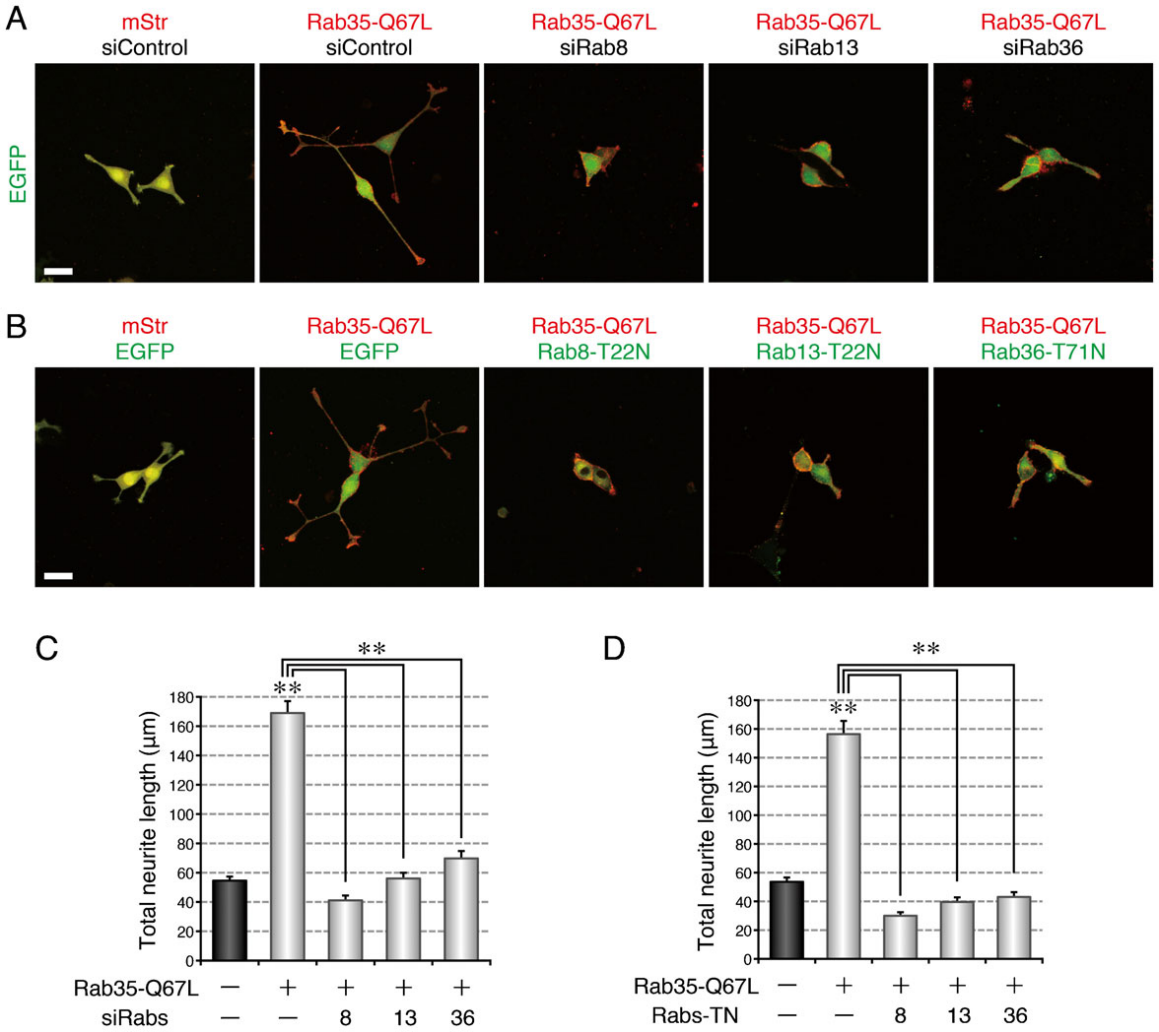
Rab8, Rab13, and Rab36 function as essential mediators for neurite outgrowth downstream of Rab35. (A) Inhibition of active Rab35-enhanced neurite outgrowth by depletion of Rab8, Rab13, or Rab36. PC12 cells expressing EGFP together with mStr + siControl, mStr–Rab35-Q67L + siControl, mStr–Rab35-Q67L + siRab8, mStr–Rab35-Q67L + siRab13, or mStr–Rab35-Q67L + siRab36 were fixed after NGF stimulation for 36 hr. (B) Inhibition of active Rab35-enhanced neurite outgrowth by expression of a dominant negative mutant of Rab8, Rab13, or Rab36. PC12 cells expressing mStr + EGFP, mStr–Rab35-Q67L + EGFP, mStr–Rab35-Q67L + EGFP–Rab8-T22N, mStr–Rab35-Q67L + EGFP–Rab13-T22N, or mStr–Rab35-Q67L + EGFP–Rab36-T71N were fixed after NGF stimulation for 36 hr. Scale bars: 30 µm. (C) Total neurite length (mean and SE) of mStr + siControl-expressing, mStr–Rab35-Q67L + siControl-expressing, mStr–Rab35-Q67L + siRab8-expressing, mStr–Rab35-Q67L + siRab13-expressing, and mStr–Rab35-Q67L + siRab36-expressing PC12 cells after NGF stimulation for 36 hr (n>100). (D) Total neurite length (mean and SE) of mStr + EGFP-expressing, mStr–Rab35-Q67L + EGFP-expressing, mStr–Rab35-Q67L + EGFP–Rab8-T22N-expressing, mStr–Rab35-Q67L + EGFP–Rab13-T22N-expressing, and mStr–Rab35-Q67L + EGFP–Rab36-T71N-expressing PC12 cells after NGF stimulation for 36 hr (n>100).

To determine whether Rab8, Rab13, and Rab36 function downstream of Rab35, we turned our attention to a constitutively active mutant of Rab35 (Rab35-Q67L), whose expression promotes NGF-induced neurite outgrowth of PC12 cells (Kanno et al., 2010; Fukuda et al., 2011). If Rab8, Rab13, and Rab36 actually function downstream of Rab35, they should be required for the promotion of neurite outgrowth by Rab35-Q67L. When we depleted PC12 cells of Rab8, Rab13, or Rab36 alone, the Rab35-Q67L-promoted neurite outgrowth was dramatically impaired (Fig. 6A,C), and the promoting effect of Rab35-Q67L on neurite outgrowth was similarly inhibited by the expression of Rab8-T22N, Rab13-T22N, or Rab36-T71N alone (Fig. 6B,D). These results taken together indicated that Rab8, Rab13, and Rab36 are essential mediators of the NGF-induced neurite outgrowth of PC12 cells that function downstream of Rab35 and MICAL-L1.

### Rab36 recruits JIP4 to recycling endosomes downstream of Rab35 and MICAL-L1 during neurite outgrowth

**Fig. 7.**
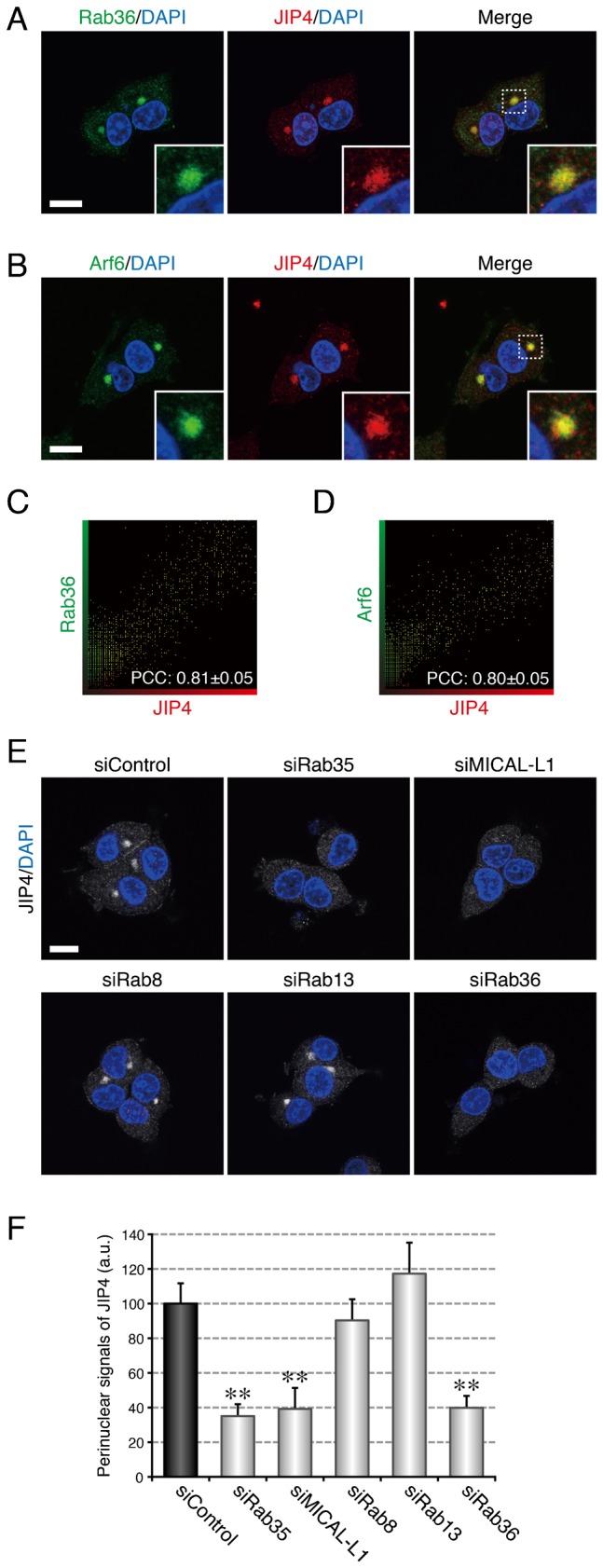
Rab36 recruits JIP4 to recycling endosomes. (A) Colocalization between Rab36 and JIP4 in PC12 cells. After NGF stimulation for 6 hr PC12 cells were fixed and stained with anti-Rab36 antibody, anti-JIP4 antibody, and DAPI. (B) Colocalization between Arf6 and JIP4 in PC12 cells. After NGF stimulation for 6 hr PC12 cells were fixed and stained with anti-Arf6 antibody, anti-JIP4 antibody, and DAPI. The insets in panels A and B are magnified views of the boxed areas. (C,D) Intensity scatter plot of Rab36 signals versus JIP4 signals (C) and of Arf6 signals versus JIP4 signals (D) in PC12 cells after NGF stimulation for 6 hr. The Pearson's correlation coefficient (PCC) value (mean ± SD) for the relation between them is shown at the bottom (n = 30 from 3 independent experiments). (E) Disappearance of JIP4 signals from the perinuclear area of Rab35-depleted, MICAL-L1-depleted, and Rab36-depleted PC12 cells. After NGF stimulation for 6 hr PC12 cells treated with siControl, siRab35, siMICAL-L1, siRab8, siRab13, or siRab36 were fixed and stained with anti-JIP4 antibody and DAPI. Scale bars: 10 µm. (F) Perinuclear JIP4 signals (mean and SE) of siControl-treated, siRab35-treated, siMICAL-L1-treated, siRab8-treated, siRab13-treated, and siRab36-treated PC12 cells after NGF stimulation for 6 hr (n = 60 from 3 independent experiments).

In the final set of experiments, in an attempt to determine whether the Rabs recruited by MICAL-L1 recruit their own effector molecules to the same perinuclear area, we turned our attention to Rab36, a previously less characterized Rab isoform, because thorough analyses of the Rab8 and Rab13 effector molecules have already been conducted (Nishimura and Sasaki, 2009; Sakane et al., 2010; Peränen, 2011). Because JIP4, a scaffold protein for kinesin-1, has been reported to localize at endosomal compartments (Montagnac et al., 2009) and is endogenously expressed in PC12 cells (supplementary material Fig. S3A), JIP4 was the most likely candidate for a Rab36 effector during neurite outgrowth among the Rab36-specific binding proteins identified thus far (Matsui et al., 2012). The results of immunofluorescence analyses showed that Rab36 colocalized with JIP4 in the perinuclear area (Fig. 7A,C) and that JIP4 also colocalized with Arf6 in the same area (Fig. 7B,D). Depletion of Rab36 by RNA interference followed by an analysis of the intracellular localization of JIP4 in NGF-stimulated PC12 cells showed a dramatic reduction in the perinuclear signals of JIP4 in Rab36-knockdown cells (Fig. 7E,F), indicating that Rab36 is required for the perinuclear recruitment of JIP4. Consistent with the fact that Rab36 is recruited to recycling endosomes by Rab35 and MICAL-L1 (Fig. 5F,G), depletion of either Rab35 or MICAL-L1 also caused a dramatic reduction in the perinuclear signals of JIP4 (Fig. 7E,F). We therefore concluded that Rab36 recruits JIP4 to recycling endosomes in PC12 cells in response to NGF stimulation only after the occurrence of the Rab35-dependent and MICAL-L1-dependent recruitment.

**Fig. 8.**
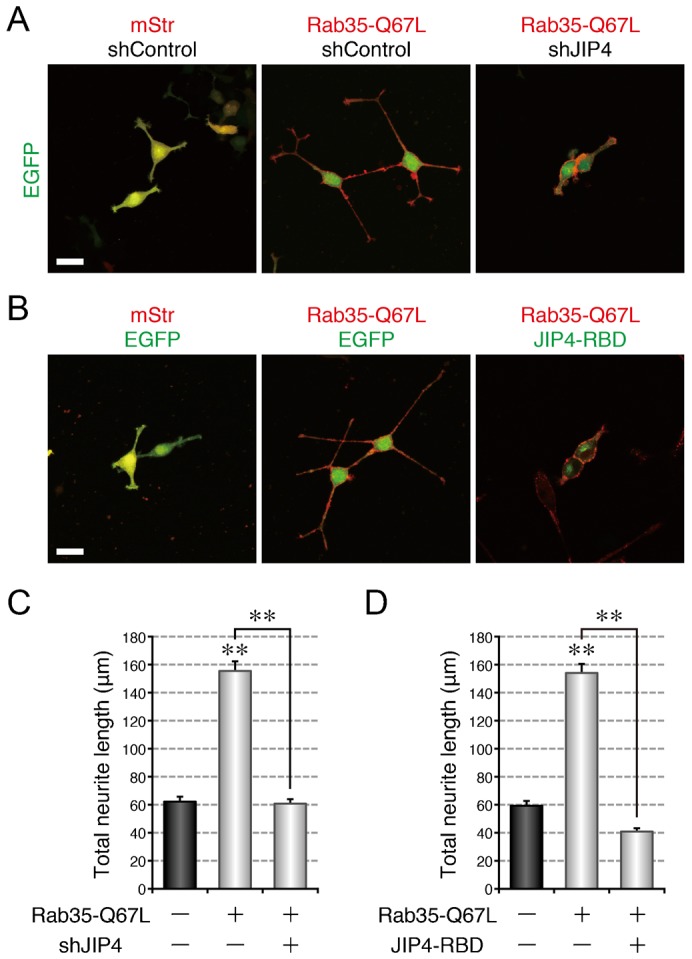
JIP4 functions as an essential mediator for neurite outgrowth downstream of Rab35. (A) Inhibition of active Rab35-enhanced neurite outgrowth by depletion of JIP4. PC12 cells expressing EGFP together with mStr + shControl, mStr–Rab35-Q67L + shControl, or mStr–Rab35-Q67L + shJIP4 were fixed after NGF stimulation for 36 hr. (B) Inhibition of active Rab35-enhanced neurite outgrowth by expression of a dominant negative mutant of JIP4. PC12 cells expressing mStr + EGFP, mStr–Rab35-Q67L + EGFP, or mStr–Rab35-Q67L + EGFP–JIP4-RBD were fixed after NGF stimulation for 36 hr. Scale bars: 30 µm. (C) Total neurite length (mean and SE) of mStr + shControl-expressing, mStr–Rab35-Q67L + shControl-expressing, and mStr–Rab35-Q67L + shJIP4-expressing PC12 cells after NGF stimulation for 36 hr (n>100). (D) Total neurite length (mean and SE) of mStr + EGFP-expressing, mStr–Rab35-Q67L + EGFP-expressing, and mStr–Rab35-Q67L + EGFP–JIP4-RBD-expressing PC12 cells after NGF stimulation for 36 hr (n>100).

Finally, we investigated whether JIP4 is an essential mediator of NGF-induced neurite outgrowth, as is Rab36 (supplementary material Fig. S2A,C). When we depleted PC12 cells of JIP4, NGF-induced neurite outgrowth of the PC12 cells was dramatically impaired (supplementary material Fig. S3A,B,D), the same as it was in the Rab36-knockdown cells. We also overexpressed the Rab36-binding domain of JIP4 (JIP4-RBD), which was expected to disrupt the interaction between Rab36 and JIP4 by masking the JIP4-binding interface of Rab36, in PC12 cells and evaluated the effect of its overexpression on neurite outgrowth. The results showed that overexpression of JIP4-RBD inhibited NGF-induced neurite outgrowth of PC12 cells (supplementary material Fig. S3C,E). Moreover, functional ablation of JIP4 either by knockdown or by JIP4-RBD overexpression dramatically inhibited the promotion of neurite outgrowth by Rab35-Q67L (Fig. 8A–D), the same as the functional ablation of Rab36 did. These results led us to conclude that JIP4 functions as a Rab36 effector molecule during NGF-induced neurite outgrowth of PC12 cells downstream of Rab35 and MICAL-L1.

## DISCUSSION

**Fig. 9.**
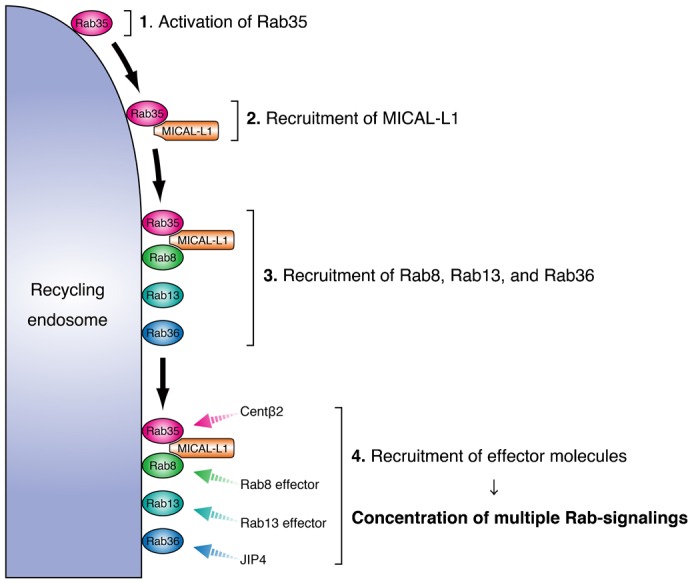
A model of Rab clustering during neurite outgrowth. In response to NGF stimulation, Rab35 (a master Rab) first accumulates at perinuclear Arf6-positive recycling endosomes. Rab35 subsequently recruits MICAL-L1 (a multiple Rab-binding molecule), which functions as a scaffold for Rab8, Rab13, and Rab36 (downstream Rabs). The downstream Rabs recruit their respective effectors, e.g. Rab36 recruits JIP4 to the same compartment, thereby resulting in the concentration of multiple Rab-signalings at the same time.

We previously reported finding that MICAL-L1, a multiple Rab-binding protein, functions as a Rab35 effector during NGF-induced neurite outgrowth of PC12 cells (Kobayashi and Fukuda, 2013a), but the significance of its multiple Rab binding activity had never been elucidated. In the present study, we for the first time demonstrated that multiple Rab binding ability of MICAL-L1, i.e. interaction with Rab8, Rab13, Rab35, and Rab36, mediates a novel multiple Rab recruitment mechanism at recycling endosomes during NGF-induced neurite outgrowth of PC12 cells. Based on these findings, we propose the following mechanism: (1) Rab35 (a master Rab) accumulates at the Arf6-positive recycling endosomes of PC12 cells in response to NGF stimulation; (2) Rab35 recruits MICAL-L1 (a multiple Rab-binding protein) to the same compartment; (3) MICAL-L1 functions as a scaffold for Rab8, Rab13, and Rab36 and recruits these downstream Rabs to the same compartment; and (4) the concentrated downstream Rabs recruit their respective effectors, e.g. Rab36 recruits JIP4, to the compartment, and we have named this multiple Rab recruitment mechanism “Rab clustering” (a schematic model is shown in Fig. 9).

Our model, in which Rab35 indirectly concentrates Rab8, Rab13, and Rab36 through MICAL-L1, seems attractive from the standpoint of explaining the efficient completion of the complicated vesicle formation step in membrane trafficking. In general, the vesicle formation step consists of the following substeps: phospholipid remodeling, coat assembly, tubulation, and scission (Mayor and Pagano, 2007; Anitei and Hoflack, 2012). Interestingly, myosin Va/b, an actin-based motor, has recently been reported to be an effector molecule of Rab8 (Roland et al., 2007; Sun et al., 2014), and JRAB, an actin regulatory protein, is known to be an effector molecule of Rab13 during neurite outgrowth (Sakane et al., 2010). Both myosin motors and the actin cytoskeleton are key regulators of fission during vesicle formation (Miserey-Lenkei et al., 2010; Liu et al., 2013; Valente et al., 2010). Similarly, a kinesin-1 motor complex has been reported to be required for tubulation during vesicle formation in addition to its conventional role in vesicle transport (Grigoriev et al., 2007). It should be noted that JIP4, which is recruited to recycling endosomes by Rab36, functions as a scaffold protein for kinesin-1 (Montagnac et al., 2009). Moreover, we previously found that Rab35 recruits centaurin-β2/ACAP2, a BAR protein that promotes tubulation, to recycling endosomes simultaneously with MICAL-L1 (Kobayashi and Fukuda, 2012; Kobayashi and Fukuda, 2013a). Thus, it is tempting to speculate that the Rabs recruited by MICAL-L1 cooperatively stimulate vesicle formation from Arf6-positive recycling endosomes. The possible involvement of Rab8, Rab13, Rab36, and their effectors in the vesicle formation step during NGF-induced neurite outgrowth is now under investigation in our laboratory.

The results of this study raised several important questions. The first question is whether the multiple Rab-binding-protein-mediated Rab recruitment is a mechanism common to all Rab family small GTPases. Because more than 20 molecules have been reported to interact with multiple Rabs (Fukuda et al., 2008; Hayes et al., 2009; Kanno et al., 2010; Lindsay et al., 2013), it is highly possible that some of them also mediate similar Rab recruitment. The second question is: Why does Rab35 alone, not Rab8, Rab13, or Rab36, function as a master Rab that determines the intracellular localization of MICAL-L1? The answer may be that the Rab that is activated first, e.g. Rab35 during neurite outgrowth, functions as a master Rab and that the master Rab varies with the cell type and/or the cellular event. Actually, our preliminary data indicated that translocation of Rab36 to the plasma membrane is also induced by Rab8-Q67L–CAAX (H.K., K.E. and M.F., unpublished data). The third question is: How are the downstream Rabs activated? Since MICAL-L1 dose not contain a GEF (guanine nucleotide exchange factor) domain, certain GEFs for Rab8, Rab13, or Rab36 must be activated before recruitment of these Rabs to Arf6-positive recycling endosomes. Whether a Rab-GEF cascade (Mizuno-Yamasaki et al., 2010) is involved in the MICAL-L1-mediated Rab recruitment is a next issue to be clarified. The fourth question is: How do the downstream Rabs promote neurite outgrowth? We have recently reported that Rab35 is translocated from Arf6-positive recycling endosomes to neurite tips during the late phase of NGF stimulation (Kobayashi et al., 2014). Since it has been proposed that recycling endosomes supply membranes and/or proteins to neurite tips, thereby enabling their outward growth (Sann et al., 2009), the downstream Rabs and their effectors may facilitate this process by promoting the formation of Rab35-posivite vesicles formed from recycling endosomes that target to neurite tips. Additional research will be necessary to answer these questions.

In summary, we have demonstrated that Rab35 recruits multiple Rabs, i.e. Rab8, Rab13, and Rab36, at recycling endosomes through MICAL-L1 during NGF-induced neurite outgrowth of PC12 cells. Our findings revealed the existence of a novel mechanism by which multiple Rabs are concentrated at the same compartment, which is triggered by a master Rab and a multiple-Rab-interacting molecule.

## MATERIALS AND METHODS

### Antibodies

Anti-Rab13 rabbit polyclonal antibody, anti-Rab15 rabbit polyclonal antibody, and anti-Rab36 guinea pig polyclonal antibody were produced by using purified GST-mouse Rab13, GST-mouse Rab15, and GST-mouse Rab36, respectively, as the antigen, and they were affinity-purified as described previously (Fukuda and Mikoshiba, 1999). The anti-Rab35 guinea pig antibody and anti-Rab36 rabbit antibody were prepared as described previously (Kobayashi and Fukuda, 2012; Matsui et al., 2012). Anti-Arf6 mouse monoclonal antibody, anti-actin goat polyclonal antibody, anti-Myc tag mouse monoclonal antibody (Santa Cruz Biotechnology, Inc., Santa Cruz, CA), anti-MICAL-L1 mouse polyclonal antibody (Abnova, Taipei, Taiwan), anti-MICAL-L1 rabbit polyclonal antibody, anti-JIP4 rabbit polyclonal antibody (Abcam K. K., Tokyo, Japan), anti-Rab8 mouse monoclonal antibody (BD Biosciences, San Jose, CA), and anti-Rab10 rabbit monoclonal antibody (Cell Signaling Technology, Beverly, MA) were obtained commercially. Horseradish peroxidase (HRP)-conjugated anti-FLAG tag (M2) mouse monoclonal antibody (Sigma–Aldrich Corp., St Louis, MO), HRP-conjugated anti-T7 tag mouse monoclonal antibody (Merck Biosciences Novagen, Darmstadt, Germany), and HRP-conjugated anti-HA tag rabbit polyclonal antibody (MBL, Nagoya, Japan) were also obtained commercially. The Alexa 488/594/633-conjugated secondary antibodies were from Invitrogen Corp. (Carlsbad, CA).

### RNA interference

Double-stranded small interfering RNAs (siRNAs) targeted to rat *Rab35* (siRab35) and rat *MICAL-L1* (siMICAL-L1) were prepared as described previously (Kobayashi and Fukuda, 2012; Kobayashi and Fukuda, 2013a). siRNAs targeted to rat *Rab8A* (siRab8A, 19-base target site: 5′-TCACGACAGCCTACTACAG-3′), rat *Rab8B* (siRab8B, 19-base target site: 5′-ATCCTTTGACAATATTAAA-3′), rat *Rab13* (siRab13, 19-base target site: 5′-GAACGATTCAAGACAATAA-3′), and rat *Rab36* (siRab36, 19-base target site: 5′-AGACTAGCCTCATTCACAG-3′) were synthesized by Nippon EGT Corp., Ltd (Toyama, Japan). Short hairpin RNAs (shRNAs) targeted to rat *JIP4* (shJIP4, 21-base target site: 5′-GGAAGTGTAATCCGTGTATAT-3′) were constructed as described previously (Kuroda and Fukuda, 2004), by using the pSilencer-neo 2.0-U6 vector (Ambion, Austin, TX), which expresses shRNA. Unless otherwise stated, throughout this paper the siRab8 for double knockdown of Rab8A and Rab8B means a mixture of siRab8A and siRab8B. Knockdown by each siRNA was confirmed by expressing it in PC12 cells for 48–60 hr and then immunoblotting with specific antibodies as described below.

### Plasmids

cDNAs encoding mouse Rab1A-Q70L, Rab1A-S25N, Rab8A, Rab8A-Q67L, Rab8A-T22N, Rab8B, Rab8B-Q67L, Rab8B-T22N, Rab10-Q68L, Rab10-T23N, Rab13, Rab13-Q67L, Rab13-T22N, Rab15-Q67L, Rab15-T22N, Rab35-Q67L, Rab35-S22N, Rab35–CAAX, Rab36, Rab36-Q116L, Rab36-T71N, MICAL-L1, MICAL-L1-CC, and JIP4 were prepared as described previously (Tamura et al., 2009; Kanno et al., 2010; Matsui et al., 2012; Kobayashi and Fukuda, 2012; Kobayashi and Fukuda, 2013a). JIP4-RBD (amino acids 474–769) was constructed by using a conventional PCR technique and the following pairs of oligonucleotides with a *Bam*HI site (underlined) or a stop codon (in bold): 5′-GGATCCAAAGACGATGATGATAGT-3′ (RBD forward primer, sense) and 5′-**TCA**ATCTAGGATGTTGCCAGG-3′ (RBD reverse primer, antisense). The resulting cDNAs were inserted into the pEGFP-C1 vector (Clontech–Takara Bio Inc., Shiga, Japan), pmStr-C1 vector, or pMyc-C1 vector (Kobayashi and Fukuda, 2012) to identify subcellular localizations and/or for neurite outgrowth assays. The resulting cDNAs were also inserted into the pEF-FLAG vector, pEF-T7 vector (Fukuda et al., 1999), or pEF-HA vector (Fukuda, 2002) for use in the coimmunoprecipitation assays. We performed DNA sequencing to confirm that no unexpected mutations had occurred in the open reading frame of the cDNAs described above.

### Cell cultures and transfections

PC12 cell and COS-7 cell culture and plasmid and/or siRNA transfection were performed essentially as described previously (Kobayashi and Fukuda, 2012).

### Immunoblotting

All of the immunoblotting procedures have been described elsewhere (Kobayashi and Fukuda, 2013a). The positions of the molecular mass markers (in kDa) are shown at the left of the immunoblotting data in the figures. The blots shown in this paper are representative of at least three independent experiments.

### Coimmunoprecipitation assays

Coimmunoprecipitation assays were performed essentially as described previously (Kobayashi and Fukuda, 2013a). In brief, lysates of COS-7 cells expressing HA–Rab35-Q67L were incubated for 1 hr at 4°C with anti-HA tag antibody-conjugated agarose beads (Sigma–Aldrich Corp.) (wet volume 10 µl). After washing the beads with 1 ml of washing buffer, the beads coupled with HA–Rab35-Q67L were incubated for 1 hr at 4°C with cell lysate expressing FLAG–Rab1A-Q70L, FLAG–Rab8A-Q67L, FLAG–Rab8B-Q67L, FLAG–Rab13-Q67L, or FLAG–Rab36-Q116L, in the absence or presence of cell lysate expressing T7–MICAL-L1. After washing the beads with the washing buffer, the FLAG–Rab1A-Q70L, FLAG–Rab8A-Q67L, FLAG–Rab8B-Q67L, FLAG–Rab13-Q67L, FLAG–Rab36-Q116L, and T7–MICAL-L1 bound to the beads were analyzed by SDS–PAGE followed by immunoblotting with HRP-conjugated anti-FLAG tag antibody and HRP-conjugated anti-T7 tag antibody. The immunoreactive bands were visualized by enhanced chemiluminescence (GE Healthcare Ltd). Input means 1/500 volume of the reaction mixture used for coimmunoprecipitation. The blots shown in this paper are representative of at least three independent experiments.

### Immunofluorescence and colocalization analyses

All of the procedures used to perform the immunofluorescence and colocalization analyses have been described elsewhere (Kobayashi and Fukuda, 2012). The results of the colocalization analyses are reported as means and standard deviation (SD) (n = 30 from 3 independent experiments for each colocalization analysis).

### Quantification of the perinuclear signals of Rab8, Rab13, Rab36, MICAL-L1, and JIP4

Perinuclear signals were quantified essentially as described previously (Kobayashi and Fukuda, 2012). In brief, 54 hr after transfecting PC12 cells with siRab35, siMICAL-L1, siRab8, siRab13, or siRab36, the cells were stimulated with NGF for 6 hr. The cells were then fixed and stained with antibody against MICAL-L1, Rab8, Rab13, Rab36, or JIP4 and fluorescence images of the transfected cells were captured at random under conditions in which none of the fluorescence signals was saturated. Because the MICAL-L1, Rab8, Rab13, Rab36, and JIP4 signals were especially concentrated around the centrosome in the perinuclear area, a 3 µm^2^ area that contained γ-tubulin signals was then selected as a region of interest (ROI), and the relative intensities of the fluorescence signals in the ROI were measured as perinuclear signals with MetaMorph software. The perinuclear signal intensity of the control cells in each experiment has been set equal to 100 (arbitrary units, a.u.). The results of the quantifications are presented as means and standard error (SE) (n = 60 from 3 independent experiments for each quantification analysis).

### Neurite outgrowth assays

Neurite outgrowth assays were performed essentially as described previously (Kobayashi and Fukuda, 2012). The results of the neurite outgrowth assays in this study are reported as means and SE of a single representative data from at least 3 independent experiments with similar results (n>100 from 3 dishes in each experiment).

### Yeast two-hybrid assays

Yeast two-hybrid assays in which pGBD-C1-Rabs (Rab1A-Q70L, Rab1A-S25N, Rab8A-Q67L, Rab8A-T22N, Rab8B-Q67L, Rab8B-T22N, Rab10-Q68L, Rab10-T23N, Rab13-Q67L, Rab13-T22N, Rab15-Q67L, Rab15-T22N, Rab35-Q67L, Rab35-S22N, Rab36-Q116L, and Rab36-T71N) were used as bait were performed essentially as described previously (Itoh et al., 2006; Fukuda et al., 2008; Fukuda et al., 2011). The cDNA of MICAL-L1-CC (coiled-coil) was also subcloned into the pACT2 vector (Clontech–Takara Bio Inc.). The yeast strain, medium, culture conditions, and transformation protocol were described previously (James et al., 1996). The results of the yeast two-hybrid assays reported in this paper are representative data from at least 2 independent experiments with similar results.

### Statistical analyses

Student's unpaired *t*-test was used to evaluate every result obtained in the experiments for statistical significance in comparison with the corresponding result obtained in the control cells. The double asterisks (**) in the bar charts indicate a *t*-test *p* value <0.01, and comparisons that yielded *p* values >0.05 are indicated by N.S. (not significant).

## Supplementary Material

Supplementary Material
