# Supplementary material for: Rab35 promotes the recruitment of Rab8, Rab13 and Rab36 to recycling endosomes through MICAL-L1 during neurite outgrowth
Source: Biol Open. 2014 Aug 1;3(9):803–14. doi: 10.1242/bio.20148771 (PMC4163657; doi:10.1242/bio.20148771)
Supplement: Supplementary Material [file supp_3_9_803__index.html]

Rab35 promotes the recruitment of Rab8, Rab13 and Rab36 to recycling endosomes through MICAL-L1 during neurite outgrowth — Supplementary Material 

# Rab35 promotes the recruitment of Rab8, Rab13 and Rab36 to recycling endosomes through MICAL-L1 during neurite outgrowth

## bio.20148771 Supplementary Material

**Files in this Data Supplement:**

- Supplementary Material - Hotaka Kobayashi et al. doi: 10.1242/bio.20148771
