# Supplementary material for: Rab35 promotes the recruitment of Rab8, Rab13 and Rab36 to recycling endosomes through MICAL-L1 during neurite outgrowth
Source: Biol Open. 2014 Aug 1;3(9):803–14. doi: 10.1242/bio.20148771 (PMC4163657; doi:10.1242/bio.20148771)
Supplement: Supplementary Material [file supp_bio.20148771_bio.20148771-s1.pdf]

Supplementary Material
Hotaka Kobayashi et al. doi: 10.1242/bio.20148771

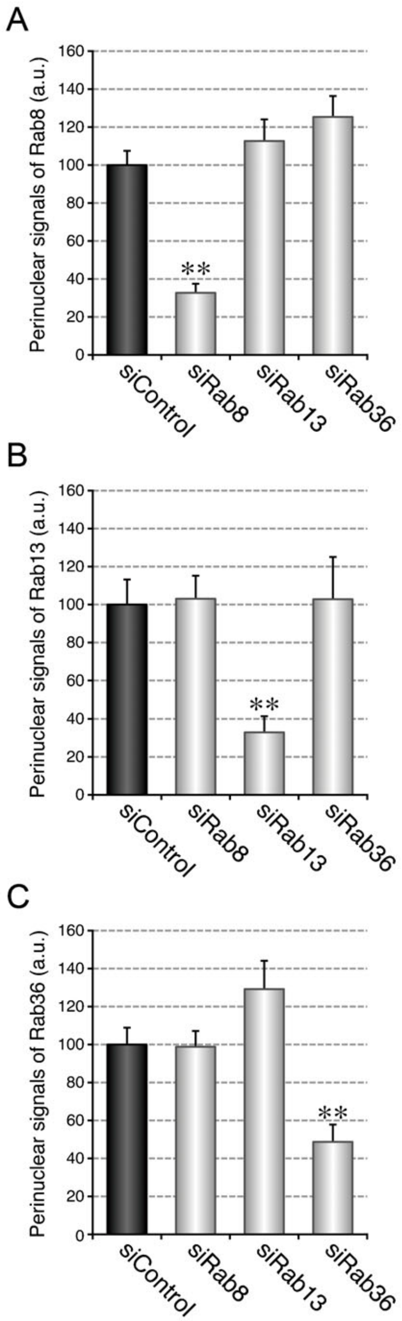

Fig. S1. Depletion of Rab8, Rab13, or Rab36 did not affect the perinuclear localization of the other downstream Rabs. Perinuclear Rab8 signals (A), Rab13 signals (B), and Rab36 signals (C) (mean and SE) of siControl-treated, siRab8-treated, siRab13-treated, and siRab36-treated PC12 cells after NGF stimulation for 6 hr (n=60 from 3 independent experiments).

Biology Open

Biology Open (2014) 000, 1–12 doi:10.1242/bio.20148771

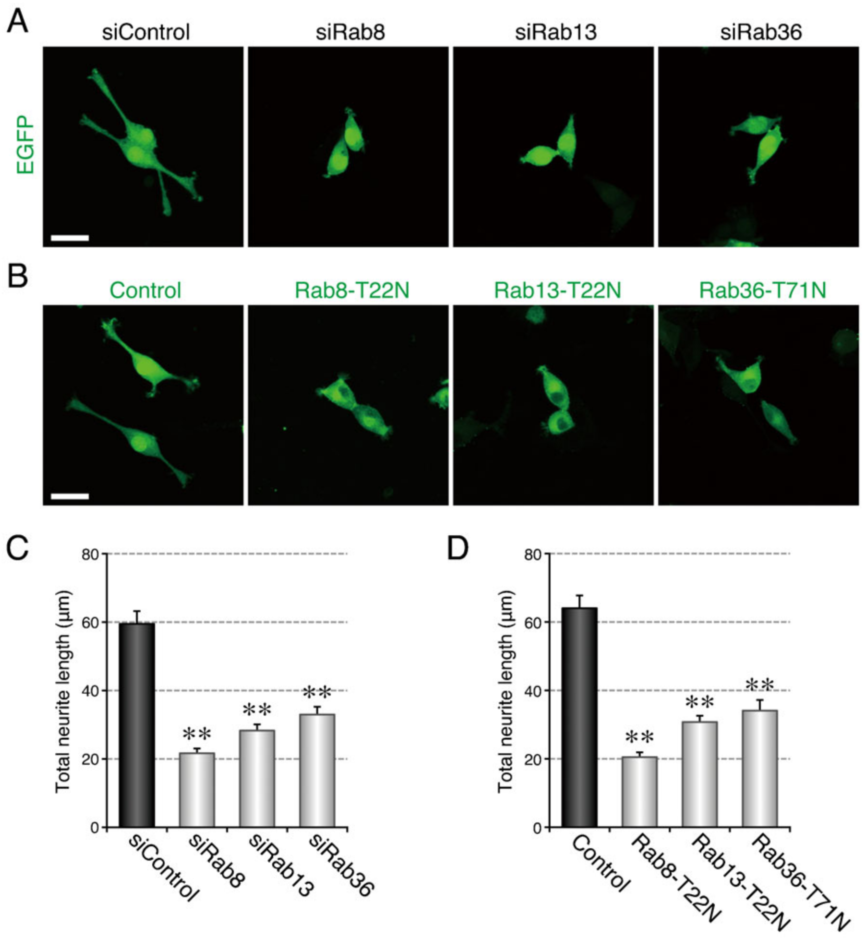

Fig. S2. Rab8, Rab13, and Rab36 function as essential mediators for neurite outgrowth.
(A) Inhibition of neurite outgrowth by depletion of Rab8, Rab13, or Rab36. PC12 cells expressing EGFP together with siControl, siRab8, siRab13, or siRab36 were fixed after NGF stimulation for 36 hr. (B) Inhibition of neurite outgrowth by expression of a dominant negative mutant of Rab8, Rab13, or Rab36. PC12 cells expressing EGFP, EGFP–Rab8-T22N (EGFP–Rab8A-T22N and EGFP–Rab8B-T22N), EGFP–Rab13-T22N, or EGFP–Rab36-T71N were fixed after NGF stimulation for 36 hr. Scale bar: 30 µm. (C) Total neurite length (mean and SE) of siControl-expressing, siRab8-expressing, siRab13-expressing, and siRab36-expressing PC12 cells after NGF stimulation for 36 hr ($n>100$). (D) Total neurite length (mean and SE) of EGFP-expressing, EGFP–Rab8-T22N-expressing, EGFP–Rab13-T22N-expressing, and EGFP–Rab36-T71N-expressing PC12 cells after NGF stimulation for 36 hr ($n>100$).

Biology Open

Biology Open (2014) 000, 1–12 doi:10.1242/bio.20148771

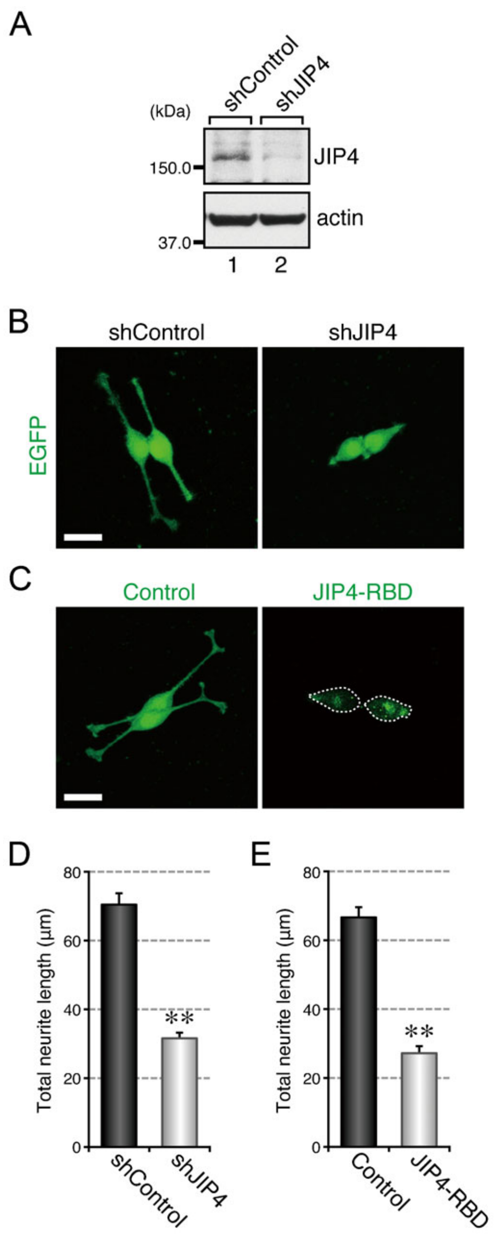

Fig. S3. JIP4 functions as an essential mediator for neurite outgrowth. (A) Reduced expression of JIP4 after treatment with shJIP4. Cell lysates of PC12 cells treated with shJIP4 were immunoblotted with anti-JIP4 antibody and anti-actin antibody. (B) Inhibition of neurite outgrowth by depletion of JIP4. PC12 cells expressing EGFP together with shControl or shJIP4 were fixed after NGF stimulation for 36 hr. (C) Inhibition of neurite outgrowth by expression of a dominant negative mutant of JIP4. PC12 cells expressing EGFP or EGFP–JIP4-RBD were fixed after NGF stimulation for 36 hr. Scale bars: 30 µm. (D) Total neurite length (mean and SE) of shControl-expressing and shJIP4-expressing PC12 cells after NGF stimulation for 36 hr ($n>100$). (E) Total neurite length (mean and SE) of EGFP-expressing and EGFP–JIP4-RBD-expressing PC12 cells after NGF stimulation for 36 hr ($n>100$).
